# Identification of Core Genes Involved in the Progression of Cervical Cancer Using an Integrative mRNA Analysis

**DOI:** 10.3390/ijms21197323

**Published:** 2020-10-03

**Authors:** Marina Dudea-Simon, Dan Mihu, Alexandru Irimie, Roxana Cojocneanu, Schuyler S. Korban, Radu Oprean, Cornelia Braicu, Ioana Berindan-Neagoe

**Affiliations:** 1 2nd Obstetrics and Gynecology Department, “Iuliu Hatieganu” University of Medicine and Pharmacy, 400012 Cluj-Napoca, Romania; marina.dudea@gmail.com (M.D.-S.); dan.mihu@yahoo.com (D.M.); 2 Department of Surgery, “Prof. Dr. Ion Chiricuta” Oncology Institute, 400015 Cluj-Napoca, Romania; airimie@umfcluj.ro; 3 Department of Surgical Oncology and Gynecological Oncology, Iuliu Hatieganu University of Medicine and Pharmacy, 400012 Cluj-Napoca, Romania; 4 Research Center for Functional Genomics, Biomedicine and Translational Medicine, Iuliu Hatieganu University of Medicine and Pharmacy, 23 Marinescu Street, 400337 Cluj-Napoca, Romania; roxana.cojocneanu@umfcluj.ro (R.C.); ioana.neagoe@umfcluj.ro (I.B.-N.); 5 Department of Natural and Environmental Sciences, University of Illinois at Urbana-Champaign, Urbana, IL 61801, USA; sskorban@illinois.edu; 6 Analytical Chemistry Department, Iuliu Hatieganu University of Medicine and Pharmacy, 4, Louis Pasteur Street, 400349 Cluj-Napoca, Romania; roprean@umfcluj.ro; 7 Department of Functional Genomics and Experimental Pathology, “Prof. Dr. Ion Chiricuţă” Oncology Institute, 34-36 Republicii Street, 400015 Cluj-Napoca, Romania

**Keywords:** cervical cancer, TCGA data, mRNA, integrative analysis

## Abstract

In spite of being a preventable disease, cervical cancer (CC) remains at high incidence, and it has a significant mortality rate. Although hijacking of the host cellular pathway is fundamental for developing a better understanding of the human papillomavirus (HPV) pathogenesis, a major obstacle is identifying the central molecular targets involved in HPV-driven CC. The aim of this study is to investigate transcriptomic patterns of HPV-infected and normal tissues to identify novel prognostic markers. Analyses of functional enrichment and interaction networks reveal that altered genes are mainly involved in cell cycle, DNA damage, and regulated cell-to-cell signaling. Analysis of The Cancer Genome Atlas (TCGA) data has suggested that patients with unfavorable prognostics are more likely to have DNA repair defects attributed, in most cases, to the presence of HPV. However, further studies are needed to fully unravel the molecular mechanisms of such genes involved in CC.

## 1. Introduction

Although cervical cancer (CC) is a preventable disease, it remains the fourth most common type of malignant cancer in women [1,2]. It accounts for higher incidence among all types of female genital tract malignant tumors, particularly detected among relatively young age groups at diagnosis [1,2]. The latest data reveal incidence of 807,860 new cases and over 200,000 deaths per year due to CC [3]. Although CC is attributed to squamous cell carcinomas (SCC) and adenocarcinomas (ADC), SCC accounts for 90% of all CC cases [3], whereas the least common subtype is that of adenosquamous carcinoma (ADSC) [4].

A large number of factors have been associated with CC, including premature onset of sexual activity, genetic background, infection, long-term administration of oral contraceptives, immunosuppressive medication, and poor health circumstances [5]. Currently, treatment for CC primarily involves surgery and radiotherapy, but sometimes chemotherapy is also used for patients with either metastasis or recurrence [6]. However, patient prognosis remains unfavorable, particularly in patients with advanced CC. Hence, it is critical that the fundamental mechanism of CC is investigated and better understood in order to develop novel therapeutic targets for CC [6], particularly those related to epithelial–mesenchymal transition (EMT). EMT is a key biological process that is involved in the activation of invasion and the metastasis of cancer cells, including those of CC [7,8,9].

Oncogenic human papillomavirus (HPV) represents an important but not a sufficient risk factor involved in the process of promoting cervical carcinogenesis [4], particularly of high-risk (HR) HPV, wherein HPV-16 and 18 are the most common types. Therefore, HPV detection is important for both the screening and prevention of cervical cancer [4]. It is reported that viral oncogenic proteins encoded by HPV are known to target and degrade the DNA repair mechanism, which, in turn, contributes to cervical cancer development [10]. Thus, hijacking of the host cellular metabolic pathway is fundamental for virus pathogenesis [11,12]. Therefore, it is critical to understand how such a hijacking mechanism recognizes central molecular targets, as well as how inputs of such targeted cellular machinery contribute to HPV-driven CC [13].

The recent boom in knowledge that shows the importance of coding and non-coding genes (particular miRNA) in the regulation of multiple major biological processes, which affect tumor genesis and tumor progression, have brought these up-until-now-neglected molecular players to the forefront [14,15,16]. The integration of high-throughput technologies and bioinformatics analysis can provide researchers with valuable data that can be exploited as biomarkers and therapeutic targets, using public data sets [17,18,19,20]. The Cancer Genome Atlas (TCGA) is a comprehensive data set that provides a unified data analysis pipeline, which can be used for additional exploration of the altered oncogenic signaling and its related implication in CC patients prognostic [21]. The scope of this study was to evaluate altered mRNA and miRNA patterns in CC, based on TCGA data following gene enrichment, to make miRNA target gene prediction and to obtain a comprehensive analysis of miRNA–mRNA regulatory networks and their correlation with overall survival of key hub transcripts.

## 2. Results

### 2.1. Differentially Expressed mRNAs and miRNA in CC Based on TCGA Data

**Figure 1:**
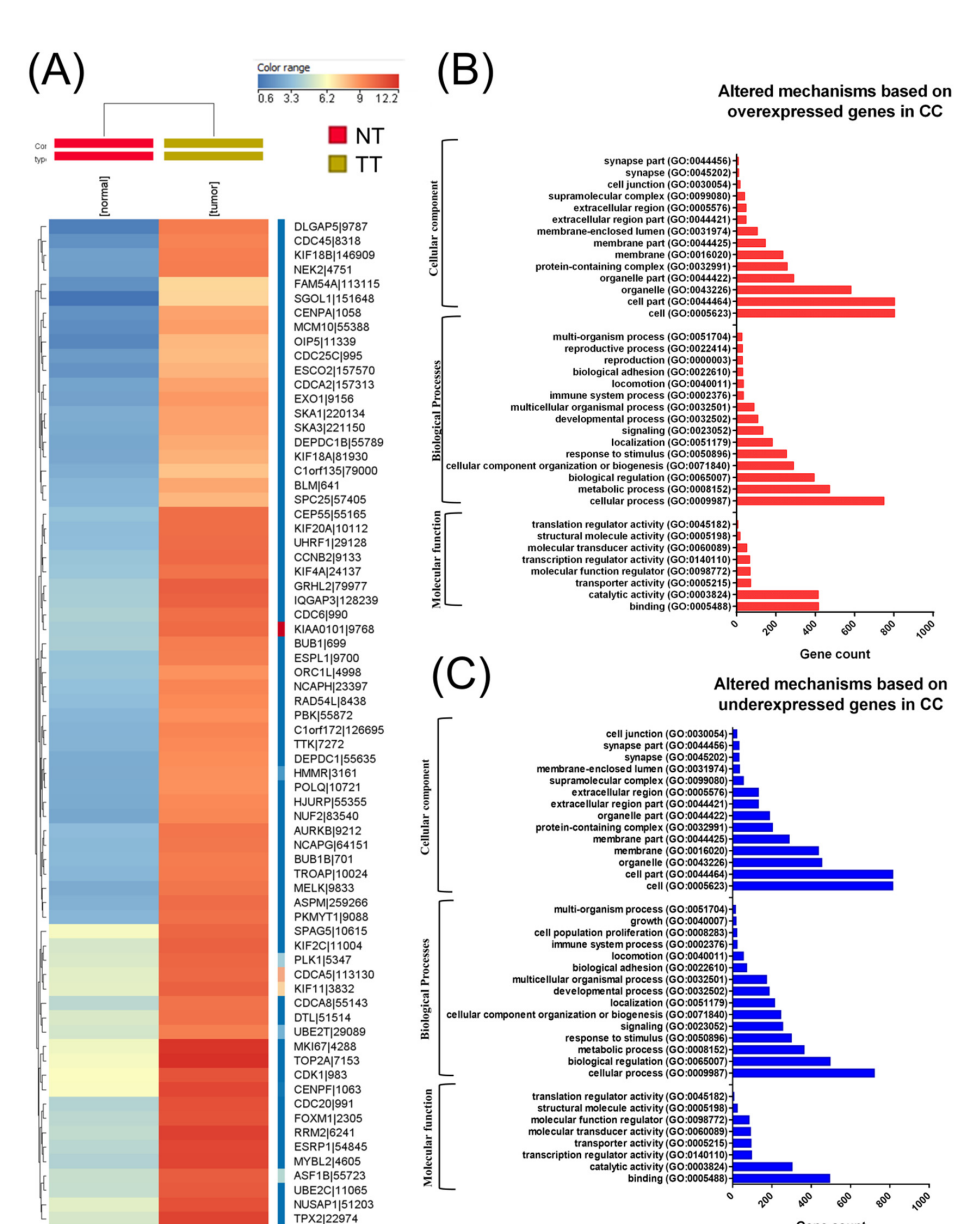
Biological interpretation of the cervical cancer (CC) altered gene expression signature. (**A**) A heatmap representation of The Cancer Genome Atlas (TGCA) gene expression data in CC, (**B**) gene ontology (GO) analysis of overexpressed genes, and (**C**) GO analysis of underexpressed genes using the Panther Gene ontology tool (available online: http://pantherdb.org); NT: normal tissue TT: tumoral tissue.

A total of 304 CC cancer samples and three analogous organ-matched normal tissues were obtained following data preprocessing. In these tissues, a total of 3988 differentially expressed RNAs were identified, wherein 2178 genes were upregulated and 1810 were downregulated (Figure 1A heatmap representation). Moreover, 207 differentially expressed miRNAs were also identified, wherein 183 miRNAs were overexpressed and 24 were underexpressed. Among altered miRNA transcripts, the top 10 overexpressed transcripts were related to EMT. Gene enrichment using Panther for those genes with altered expression levels in CC is presented in Figure 1B,C.

### 2.2. Functional Enrichment Analysis

**Figure 2:**
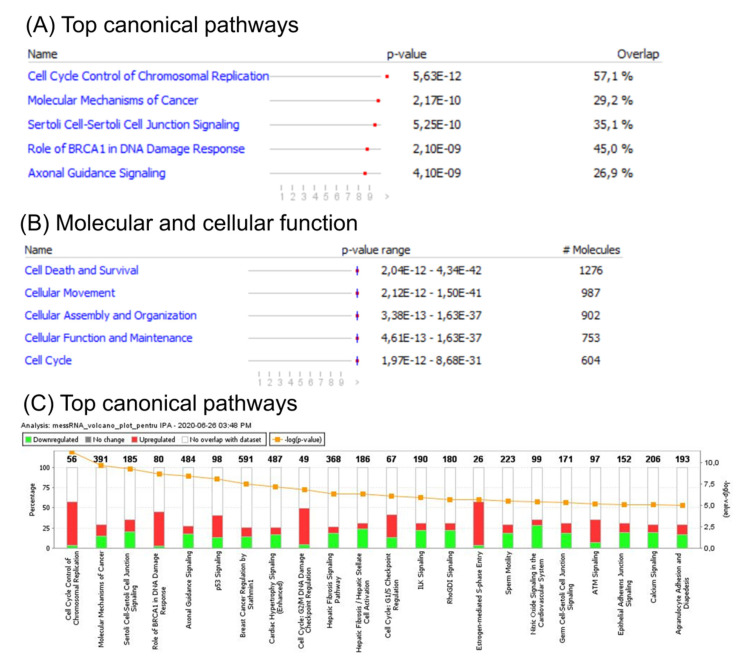
Functional analysis of mRNA altered genes in CC. (**A**) Top canonical pathways; (**B**) top molecular function; (**C**) ingenuity pathway analysis (IPA) of differentially expressed pathways specific to CC. A log Benjamini–Hochberg value higher than 5, *p*-value ≥ 0.0001, corresponds to a significantly altered pathway. The solid yellow line in the bar graph corresponds to ratios of number of molecules from the dataset that map to the pathway over the total number of molecules that map to the canonical pathway from the IPA knowledge base. The Z score denotes the activation score for either a pathway, disease, or function. This score corresponds to levels of activation (red) or deactivation (green) of a pathway or function due to changes in expression of genes involved in pathways or functions.

An ingenuity pathway analysis (IPA) of the canonical signaling pathway, for disease and function, was subsequently performed to identify enrichment of genes altered in CC. This analysis revealed involvement of several cell cycle related pathways, including G2/M DNA damage check-point regulation, G1/S check-point regulation, and BRCA1 DNA damage response. Additional altered signaling related to either TP53 signaling or to those related to both cellular adhesion and tight junction proteins was observed. Gene enrichment analysis of differentially expressed genes revealed the most important altered canonical pathways (Figure 2A), top molecular function (Figure 2B), and the most specific differentially expressed pathways related to CC (Figure 2C), based on mRNA patterns.

### 2.3. Gene Network Analysis

In addition to the delineation of main pathways and cellular functions, gene networks have been constructed to link key genes and enriched categories of diseases and functions based on associations between differentially expressed genes. These gene networks along with their top associated diseases and functions are presented in Table 1.

**Figure 3:**
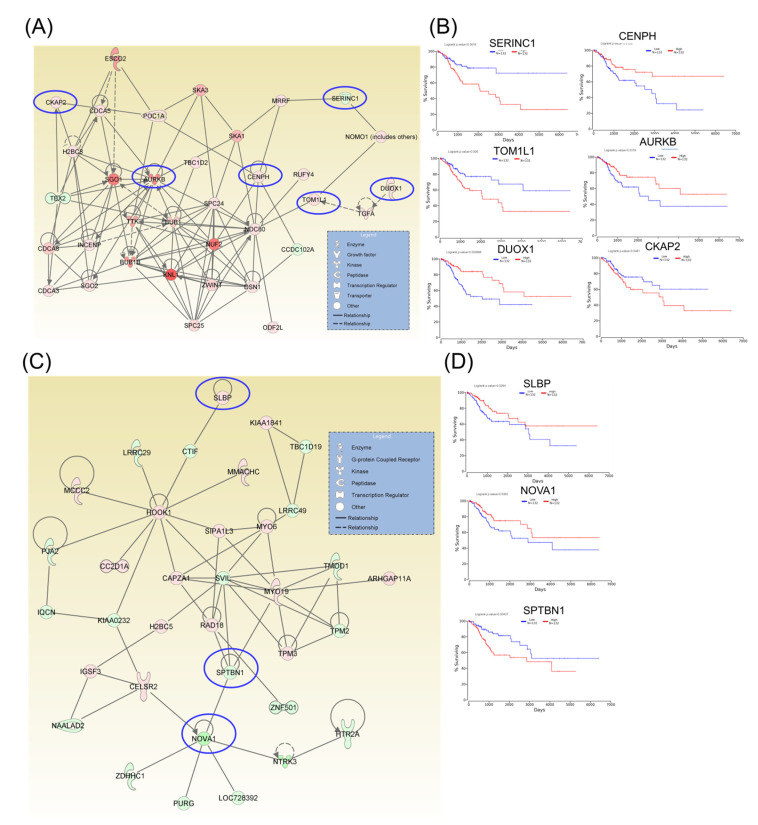
A signaling network analysis using the IPA software for differentially expressed genes in CC. (**A**) Network 1: Cell Cycle; Cellular Assembly and Organization; and DNA Replication, Recombination, and Repair. (**B**) Genes predicting overall survival in network 1. (**C**) Network 2: Cellular Assembly and Organization. (**D**) Genes predicting overall survival in Network 2; red: overexpressed genes, green: downregulated genes, blue circles are for genes predicting overall survial rate in CC

**Figure 4:**
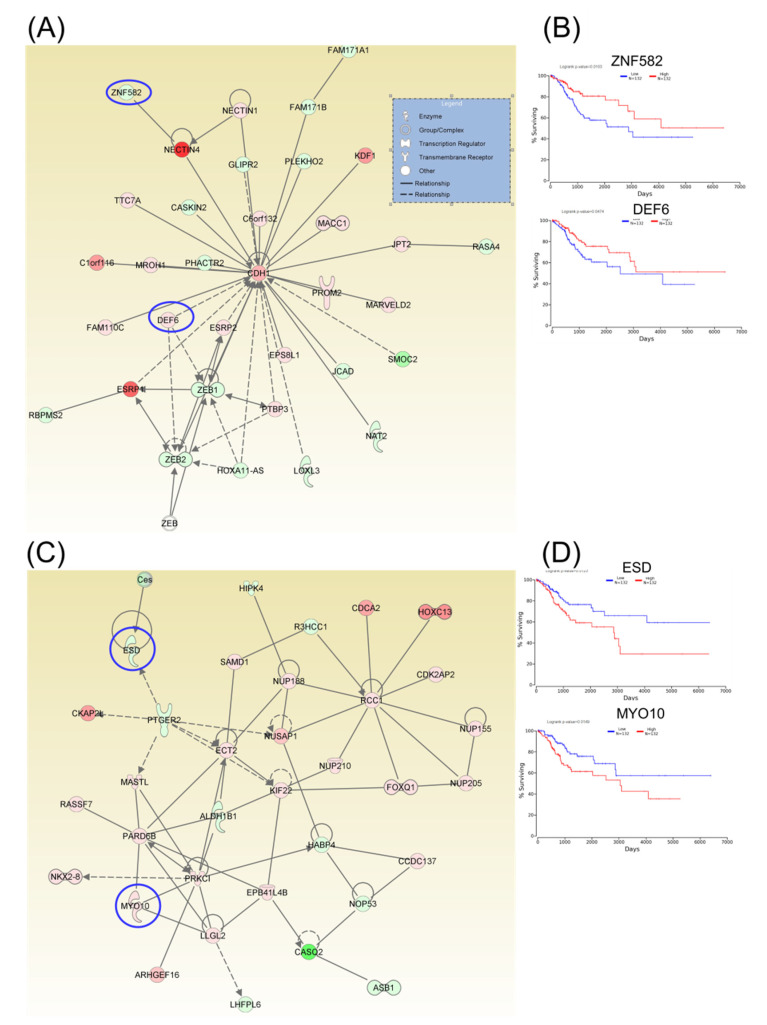
A signaling network analysis using IPA software for differentially expressed genes in CC. (**A**) Network 7: Cell-To-Cell Signaling and Interaction, Cellular Assembly and Organization, as well as Cellular Development. (**B**) Genes predicting overall survival in network 7. (**C**) Network 8: Cell-To-Cell Signaling and Interaction, Cellular Assembly and Organization, as well as Cellular Development. (**D**) Genes predicting overall survival in Network 8; red: overexpressed genes, green: downregulated genes, blue circles are for genes predicting overall survial rate in CC

Following identification of hub genes that might be involved in CC progression, a Kaplan–Meier plotter (http://www.oncolnc.org) was performed to identify those genes with significant effects. As a result, six hub genes for survival (SERINC1, TOM1L1, DUOX1, CENPH, AURKB, and CKAP2) related to cell cycle, cellular assembly and organization, DNA replication, recombination, and repair, as well as another three hub genes for survival (SLBP, NOVA1, and SPTBN1) related to cellular assembly and organization, were identified, and these genes were graphically presented using OncoLnc (Figure 3). Similarly, two additional hubs (networks 7 and 8) were observed for overall survival for genes ZNF582 and DEF6, as well as for genes ESD and MYO10, respectively (Figure 4).

**Figure 5:**
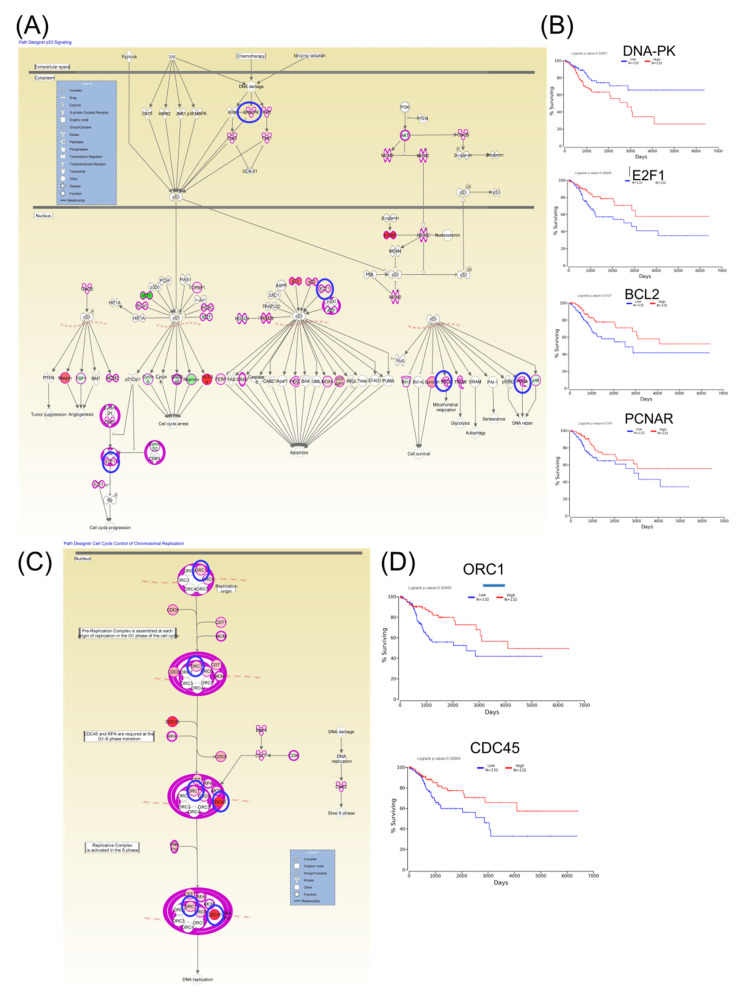
An IPA gene interconnected network based on altered genes in CC. (**A**) IPA generated networks focusing on altered genes in CC related to TP53 signaling. (**B**) Genes predicting overall survival related to TP53 signaling. (**C**) IPA generated networks focusing on altered genes in CC related to cell cycle control of chromosomal replication (**D**) Genes predicting overall survival related to cell cycle control of chromosomal replication; overexpressed genes are displayed in red, while downregulated are in green; blue circles are for genes predictin overall survial rate in CC

**Figure 6:**
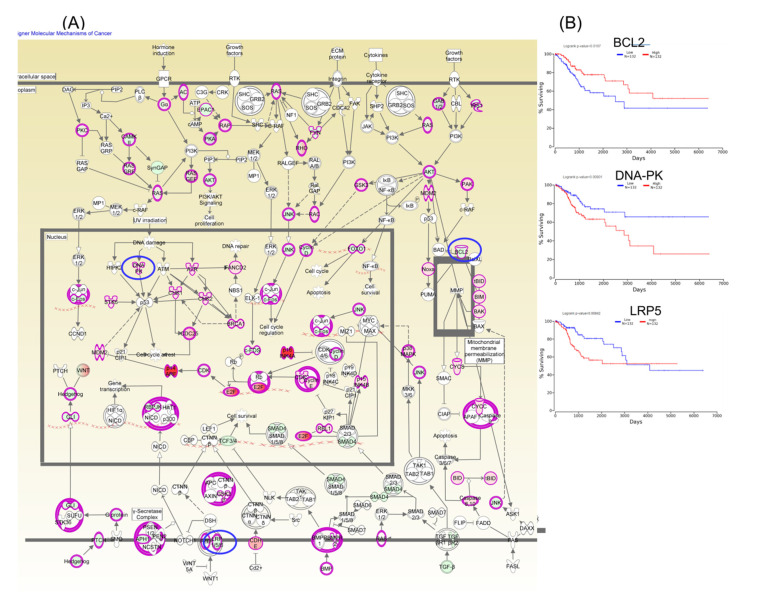
A molecular mechanism for cancer pathogenesis based on altered genes in CC (Panel **A**), wherein the key genes BCL2, DNA-PK, and LRP5 predict overall survival rate (Panel **B**); red: overexpressed genes, green: downregulated genes, blue circles are for genes predicting overall survial rate in CC.

Furthermore, Kaplan–Meier plotter online tools were used to identify prognostic information related to gene networks of differentially expressed genes involved in TP53 signaling (Figure 5). This revealed that four genes (DNA-PK, E2F1, BCL2, and PCNAR) correlated well with overall survival, cell cycle control, and chromosomal replication, while two other genes (ORC1 and CDC45) correlated with overall survival. As for altered genes related to molecular mechanisms for cancer (Figure 6), a total of six genes from network 1 and three genes from network 2 were correlated with overall survival.

**Figure 7:**
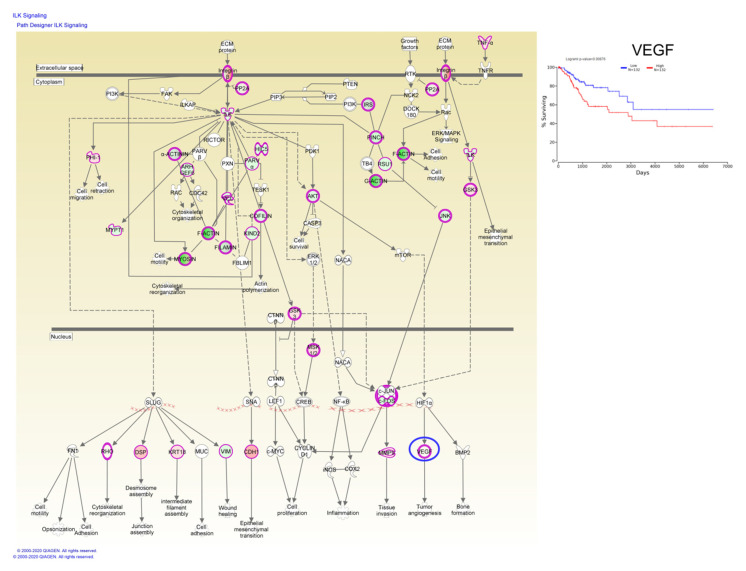
ILK signaling based on altered genes in CC, wherein the VEGF gene predicts the overall survival rate; red: overexpressed genes, green: downregulated genes, blue circles are for genes predicting overall survial rate in CC.

Taken together, these data have demonstrated that these candidate genes are undoubtedly associated with the prognosis of CC patients, whereas, Kaplan–Meier plotter online tools have identified a single gene, vascular endothelial growth factor (VEGF), involved in ILK signaling capable of predicting overall survival rate (Figure 7).

### 2.4. mRNA-miRNA Interactions in CC.

**Figure 8:**
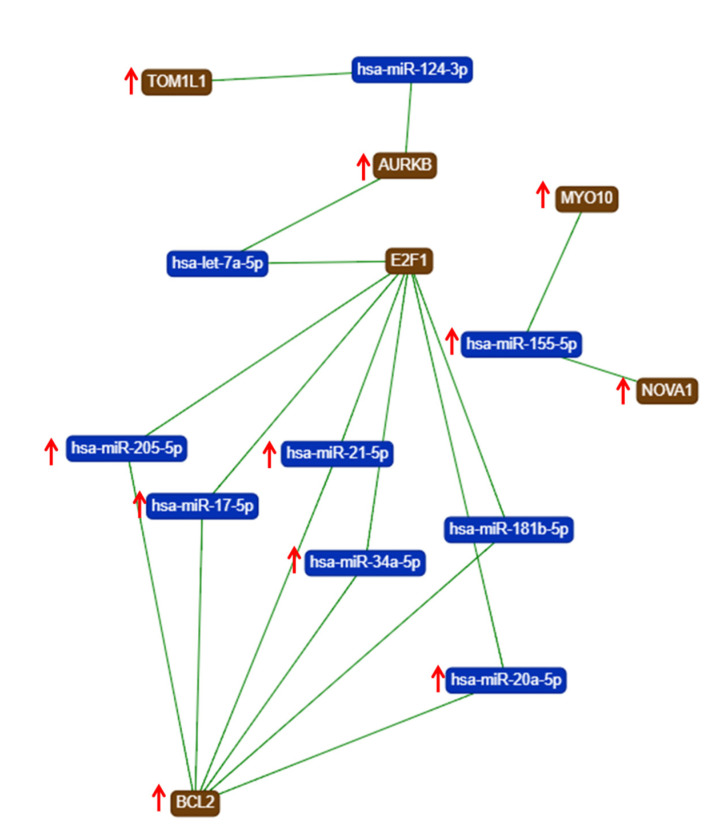
An mRNA–miRNA iinteraction map for each of the hub genes with prognostic values for CC, generated using miRtargelink (Available online: https://ccb-web.cs.uni-saarland.de/mirtargetlink/). Only those strong interactions are herein presented (↑ overexpressed transcript).

**Figure 9:**
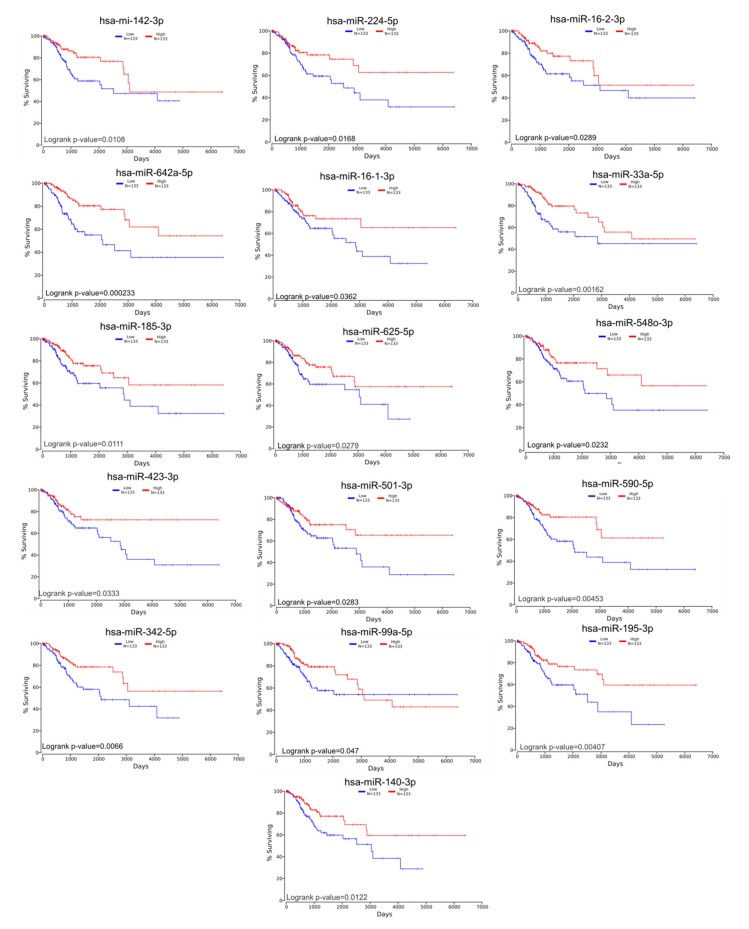
Altered miRNAs in CC predicting overall survival rate.

**Figure 10:**
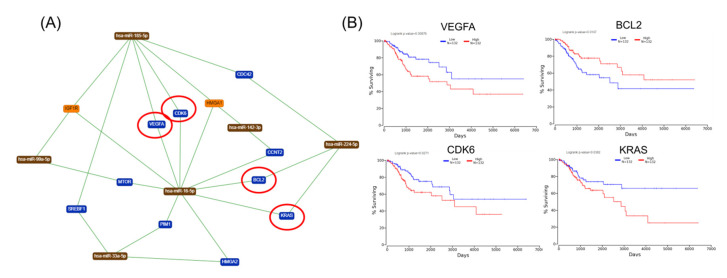
mRNA-mRNA network interaction in CC. (**A**) An mRNA–miRNA interaction map based on miRNAs predicting overall survival in CC, generated using miRtargelink (Available online: https://ccb-web.cs.uni-saarland.de/mirtargetlink/). Only those strong interactions are herein presented; (**B**) network genes predicting overall survival rate in CC; red circles are for genes predicting overall survial rate in CC.

The gene list with prognostic values in CC was used for assessing interactions with miRNA using miRtargetlink online software. This represented only the strong connection nodes. It was found that BCL2 and E2F1 genes were connected with key overexpressed altered transcripts (miR-205-5p, miR-17-5p, miR-21-5p, miR-34a-5p, and miR-20a-5p) (Figure 8). Furthermore, it was observed that the multifunctional miR-155 targeted both Nova1 and Myo10. However, none of these miRNAs were capable of predicting the overall survival rate in CC, although it was observed above that miRNAs were capable of predicting overall survival rate in CC (Figure 9). When performing analysis for interconnectedness of miRNAs predicting overall survival rate, it was found that these transcripts targeted the key genes BCL2, VEGFA, and KRAS (Figure 10).

## 3. Discussion

Although earlier studies have reported on progress in elucidating the potential molecular mechanism of CC development [22,23], the fundamental knowledge of this mechanism remains problematic. As HPVs are DNA viruses with epithelial tropisms, HPV infection results in activation of the DNA damage repair mechanism [13]. It has been reported that activation of factors related to DNA damage correlates with CIN progression [24]. As of now, there is no targeted therapy; therefore, DNA damage-related genes may serve as novel biomarkers for predicting those CC patients who are more likely to have a poorer prognosis [10,25].

Previous studies have described an abnormal regulation of the cell cycle mechanism that plays a vital role in both tumorigenesis and progression, including that detected in CC [26]. In this study, a more complex alteration mechanism at both mRNA and miRNA is observed. This interconnected mechanism is capable of identifying some key hub genes with prognostic values in CC. This interconnection among the prognostic mRNA-miRNA is presented in Figure 10.

In recent years, DNA damage and repair have increased in interest, yielding opportunities for exploring the mechanistic basis underlying potential therapeutic vulnerabilities [12,27,28,29,30]. Therapeutic targeting of these repair constituents may be uniquely advantageous in CC, particularly to be most effective in combination with standard therapy. For example, it has been reported that overexpression of AURKB is correlated with poor clinical prognosis in CC patients, suggesting its interference with a vital function, and thus serving as a potential therapeutic target [31].

A distinct aspect of the initiation of tumorigenesis is the case of HPV negative CC patients. Indeed, in recent years a subset of HPV-negative CC has been highlighted, thus contradicting the widely accepted hypothesis that HPV infection is necessary for the onset of CC [32]. These cancers are more commonly non-squamous, and represent rare subtypes [32]. As the pathogenetic mechanism for tumorigenesis may differ in these forms, a broader and more comprehensive analysis should include a significantly higher numbers of patients since the current numbers of reported cases are rather limited. Therefore, it would be valuable to analyze potential triggers of carcinogenesis in non-HPV CC.

Although TOM1L1 regulates the oncoprotein ERBB2-driven cell invasion with metastatic phenotypes in breast cancer [33], there is no available information on the role of this gene in CC. Another gene predicting overall survival is DOXO1 (Dual oxidase 1), whose overexpression has been reported to promote favorable effects in CC patients via immune response activation [34]. In this study, we have observed that increased expression levels for this dual gene are related to a better prognostic.

Furthermore, the association of HPV infection with overexpression of both TP53 and BCL2 proteins in pre-malign lesion of the uterine cervix has been previously reported [35]. In contrast in this study, we have noted inhibition of BCL2 expression, whereas BCL2 overexpression is observed to be associated with better prognostic [36,37]. Furthermore, in this study, we have observed that high expression levels of BCL2 predict a better survival rate.

It is well known that cell cycle genes play important roles in cell proliferation, as well as in cell growth [24,25]. In this study, it is revealed that alteration of cell cycle signaling pathways has allowed for identification of important candidate genes related to cell cycle loss of control, thus promoting CC pathogenesis, as it has been previously reported for both CDC45 and ORC1 [38]. CDC45 plays a key role in late G1, as it is a key element of the DNA replication machinery; thus, it is deemed as a key element of the pathogenic network in CC [38]. Furthermore, DNA-PK, a dynamic enzyme involved in DNA double-stranded breaks repair pathway [39], has been proposed as a therapeutic target [40,41], while E2F1 plays critical roles in both cell cycle regulation and chromosome segregation. It has been reported that E2F1 may promote DNA replication and cancer cell proliferation. Furthermore, both viral E6 and E7 decrease NOVA1 expression, but only E7 increases expression of RNASEH2A in an E2F1-dependent manner [42]. Therefore, all these genes are worthy of further testing for their roles in HPV infection, including tumor initiation and CC progression [43]. In this study, we have observed that CDC45, DBA-PK, E2F1, and NOVA1 are expressed in CC patients, and therefore, they can be used as prognostic markers for CC.

In this study, CKAP2 has been observed to have an oncogenic function in CC development, based on TCGA data, and this is likely accomplished via its interference with FAK-ERK2 signaling [44]. Another gene detected in this study to be involved in CC pathogenesis is DUOX1. This gene plays a significant role in host mucosal immunity by producing hydrogen peroxide, thus serving as the first line of defense to HPV invasion, particularly in cervical cancer [34]. Yet another critical gene identified in this study is VEGF. The expression pattern of this gene in CC patients suggests that VEGF is mainly related to the response to neoadjuvant chemotherapy, and to an unfavorable prognosis [11,45]. Therefore, the proangiogenic factor VEFG is not only an important modulator of immune responses but it also interferes in key oncogenic signaling in CC, as angiogenesis is a key mechanism activated in CC [25].

Biologically significant major genes related to CC are summarized in Table 2 (all these genes can be included in further studies for validation as biomarkers). These should not be considered as individual genes, but as a part of the complex miRNA–mRNA interconnections, as can be observed in Figure 10. VEGFA, a proangiogenic factor, and BCL2, a key apoptosis regulator, should be considered as therapeutic targets.

## 4. Materials and Methods

### 4.1. TCGA Data Collection

RNAseq data and corresponding clinical data of 304 cervical squamous cell carcinoma (CESC) samples and three analogous organ-matched normal tissues samples were downloaded from The Cancer Genome Atlas (TCGA) database (http://firebrowse.org/). All clinical information for patients included in this study is presented in Table 3.

### 4.2. Differentially Expressed Analysis and Survival Analysis

Identification of differentially expressed genes and miRNAs was performed using GeneSpring version 14.5 (Agilent Technologies, Santa Clara USA). Thresholds for both gene expression and miRNA analysis were as follows: fold-change (FC) ±2 and false discovery rate (FDR) ≤ 0.05.

### 4.3. OncoLnc

OncoLnc is a Kaplan–Meier plotter tool, accessible online, commonly used to link TCGA survival data to mRNA, miRNA, and lncRNA expression levels (http://www.oncolnc.org). The log rank value and hazard ratio (HR) with 95% confidence intervals and 50% percentile were computed, and presented along the plot.

### 4.4. Gene Enrichment Analysis

Screened altered genes in CC were submitted to the PANTHER online tool (http://www.pantherdb.org) for displaying Gene Ontology (GO) classifications of these genes based on molecular function, biological processes, and cellular function.

### 4.5. Gene Network analysis

A gene network analysis was performed using the Ingenuity Pathway Analysis (IPA) software (Ingenuity Systems, Redwood City, CA, USA). All altered genes in CC networks were algorithmically generated based on their connectivity, and scores were assigned. Each score was presented as a numerical value, based on the relevance of altered genes overlapping with the database and taking into account relevance of this network to the original list of target genes. A canonical pathways analysis identified the most significant pathways, from the IPA library of canonical pathways, which were most relevant based on the input dataset.

## 5. Conclusions

In this study, analysis of TCGA has revealed that patients with unfavorable prognostics are more likely to have DNA repair defects, and that in most cases, this is most likely due to presence of HPV. Furthermore, key genes with prognostic values involved in CC are identified. These genes could be potentially used in pursuing molecular diagnosis or treatment of CC. However, additional studies should be conducted to fully decipher the molecular mechanisms of these key genes involved in CC.

## Figures and Tables

**Table 1:** List of the top 10 altered networks in CC based on IPA analysis.

Network	Top Diseases and Functions	Score	Focus Molecules	Molecules in a Network
Network 1	Cell Cycle; Cellular Assembly and Organization; DNA Replication, Recombination, and Repair	32	35	AURKB,BUB1,BUB1B,CCDC102A,CDCA3,CDCA5,CDCA8,CENPH,CKAP2,DSN1,DUOX1,ESCO2,H2BC8,INCENP,KNL1,MRRF,NDC80, NUF2, ODF2L,POC1A, RUFY4, SERINC1, SGO1,SGO2,SKA1,SKA3, SPC24, SPC25, TBC1D2,TBX2,TGFA,TOM1L1,TTK ZWINT
Network 2	Cellular Assembly and Organization, Developmental Disorder, Skeletal and Muscular Disorders	32	35	ARHGAP11A,CAPZA1,CC2D1A,CELSR2,CTIF,H2BC5,HOOK1,HTR2A,IGSF3,IQCN,KIAA0232,KIAA1841,LOC728392,LRRC29,LRRC49,MCCC2,MMACHC,MYO19,MYO6,NAALAD2,NOVA1,NTRK3,PJA2,PURG,RAD18,SIPA1L3,SLBP,SPTBN1,SVIL,TBC1D19,TMOD1,TPM2,TPM3,ZDHHC1,ZNF501
Network 3	Cardiovascular Disease, Congenital Heart Anomaly, Developmental Disorder	32	35	AGAP5,ATP1A1,ATR,AVPR2,BEX4,BHMT2,C12orf57,C16orf89,CITED2,DCUN1D1,EEF1A1,ELFN1,FBXO31,HSPA1L,HSPB2,IDH2,KLHL13,LGALS7/LGALS7B,MAOB,MARK2,MRPL12,MTHFS,NUAK2,NUDT1,PITX2,PKD1,PPM1K,PPP1CA,PPP1R12A,PPP1R12C,SLC45A1,TFAP2A,TMEM132C,TPT1,TUBG1
Network 4	Connective Tissue Disorders, Developmental Disorder, Organismal Injury and Abnormalities	32	35	ACTL6A,ADRA1D,ARMCX5-GPRASP2/GPRASP2, C10orf25,CCT5,EFTUD2,EIF3E,H1-2, HSPH1, KIFC2,KLHL33,LRRC59,MCM2, MCM5, MNX1, NAALADL1, NCBP1, OPLAH, PGAM5, PRKDC, RECQL4, RIPK4, RPL3, RUVBL1,SDC2,SIRT7,SMC1A,SPACA9,TCAM1P,TCOF1,TMPO,WDR86, ZC3H6,ZC3H8,ZFP69B
Network 5	Cell Morphology, Connective Tissue Disorders, Hereditary Disorder	30	34	ANO2,CENPI,CENPK,CENPL,CENPM,CENPN,CENPO,CENPP,CENPQ,CENPU,CENPW,CLIC3,DCBLD1,ESR1,FGD5,MND1,Mta,ODF3B,PCDH12,PCDHB4,PCDHB6,PCDHGA11,PLXDC1,PSD4,RAI2,RERG,SEC22C,SSR4P1,TESMIN,TSPAN9,TTC9,TTLL11,UBL3,ZNF366,ZNF367
Network 6	Embryonic Development, Nervous System Development and Function, Organ Development	30	34	ARHGAP31,C19orf33,C1QTNF9,C1QTNF9B,CHN2,CIP2A,CNPY4,COBLL1,COL6A2,COLGALT2,CWF19L2,CYSRT1,DSC2,FAM110D,FAM117A,GYG1,KERA,LUM,MFAP5,MICOS10-NBL1/NBL1,MTFR1L,OSCP1,P3H3,PITPNM1,PODN,Rac,ROBO4,RSRC1,SH3BP1,SLC12A8,SLIT2,SLIT3,TMC4,TMEM245,TRIM7
Network 7	Cell-To-Cell Signaling and Interaction, Cellular Assembly and Organization, Cellular Development	30	34	C1orf116,C6orf132,CASKIN2,CDH1,DEF6,EPS8L1,ESRP1,ESRP2,FAM110C,FAM171A1,FAM171B,GLIPR2,HOXA11-AS,JCAD, JPT2,KDF1,LOXL3, MACC1, MARVELD2, MROH1,NAT2,NECTIN1, NECTIN4, PHACTR2,PLEKHO2,PROM2,PTBP3,RASA4,RBPMS2,SMOC2,TTC7A,ZEB,ZEB1,ZEB2,ZNF582
Network 8	Cell Signaling, Infectious Diseases, Post-Translational Modification	30	34	ALDH1B1,ARHGEF16,ASB1,CASQ2,CCDC137,CDCA2,CDK2AP2,Ces,CKAP2L,ECT2,EPB41L4B,ESD,FOXQ1,HABP4,HIPK4,HOXC13,KIF22,LHFPL6,LLGL2,MASTL,MYO10,NKX2-8,NOP53,NUP155, NUP188,NUP205, NUP210, NUSAP1,PARD6B, PRKCI,PTGER2, R3HCC1,RASSF7,RCC1,SAMD1
Network 9	Cellular Development, Cellular Growth and Proliferation, Embryonic Development	30	34	ADGRE5,ADGRG1,ATOH8,CNTF,CYS1,DENND2A,EID2B,EPO,FANCC,FZD4,FZD6,GATA2,GIPR,LMO2,MFSD13A,MFSD2B,N4BP2L1,NDN,NPY1R,NR4A3,PRL,Proinsulin,PTGFR,RUNX1,SLC24A3,SLC4A11,SLC9B2,SOBP,TACSTD2,TAL1,TFRC,TPSG1,TXNIP,VPS51,ZNF788P
Network 10	Developmental Disorder, Molecular Transport, Protein Trafficking	30	34	ASPA,ASTN1,CGAS,CSE1L,DENND5B,DNMT1,ESPL1,FGD3,Flotillin,FOXA1,GATA6,HAPLN2,HBP1,HDAC1,HTR2B,KPNA2,MACROH2A1,MCM3,PHF21B,PLEKHH1,POP1,PSMA6,RAN,SERPINB2,SLX1A/SLX1B,SORBS3,STXBP5L,TCF20,TCF7L2,TDRKH,TRAM1L1,USHBP1,VAMP5,VXN,ZNF25

**Table 2:** Major functions of important prognostic genes related to CC.

Gene Symbol	Gene Nomenclature	Expression Level	Gene Function	Specificity According to Protein Atlas	Therapeutic Value and Utility	References
AURKB	Aurora Kinase B	Up	mitosis and cytokinesis	Lower cancer specificity	Prognostic and therapeutic target	[31]
BCL2	B-cell lymphoma 2	Up	Apoptosis	Lower cancer specificity	Prognostic and therapeutic target; overexpression favorable prognostic	[36,37]
CDC45	Cell division cycle protein 45	Up	Cell cycle	Lower cancer specificity	Prognostic	[38]
CENPH	Centromere protein H	Up	centromere complex	Lower cancer specificity	-	[46]
CKAP2	Cytoskeleton-associated protein 2	Up	Cell cycle and cell death	Lower cancer specificity	-	[47]
DNA-PK	Protein kinase, DNA-activated, catalytic polypeptide	Up	DNA repair	Lower cancer specificity	Therapeutic target	[39,40]
DUOX1	Dual oxidase 1	Up	ROS	Cancer enhancer (thyroid cancer)	Prognostic marker and therapeutic target; overexpression favorable prognostic;	[34]
E2F1	E2F Transcription Factor 1	Up	Cell cycle regulation	Lower cancer specificity	Overexpression favorable prognostic;	[42,43]
NOVA1	Neuro-oncological ventral antigen 1	Up	mRNA processing	Cancer-enriched (glioma)	-	[42]
ORC1	Origin Recognition Complex Subunit 1	Up	Cell cycle	Lower cancer specificity	-	[48]
PCNAR	Proliferating cell nuclear antigen	Up	Cell cycle	-	-	-
SERINC1	Serine incorporator 1	Down	-	Lower cancer specificity	-	-
SLBP	Stem-loop binding protein	Up	Cell cycle regulation	Lower cancer specificity	-	-
SPTBN1	Spectrin beta, non-erythrocytic 1	Up	--	Lower cancer specificity	-	-
TOM1L1	Target of myb1 like 1 membrane trafficking protein	Up		Lower cancer specificity	-	-
VEGFA	Vascular endothelial growth factor	Up	Angiogenesis	Lower cancer specificity	Prognostic marker; increased expression worse prognosis	[11,45,49]

- data not avalilable.

**Table 3:** Clinical data for cervical squamous cell carcinoma (CESC) patients (TCGA).

Demographics		CESC (*n* = 304)
Age	Median, Range ♀	46, 20–88
HPV Status	Positive	281
	Negative	22
	Indeterminate	1
Pathologic TNM	T1	140
	T2	71
	T3	20
	T4	10
	Tis	1
	Tx	17
	T unknown	45
	N0	133
	N1	60
	Nx	66
	N unknown	45
	M0	116
	M1	10
	Mx	128
	M unknown	50
Clinical stage	I	162
	II	69
	III	45
	IV	21
	Unknown	7
Birth control pill use	Current user	15
	Former user	53
	Never used	89
	NA	147
Histological type	Adenosquamous	5
	Cervical squamous cell carcinoma	252
	Endocervical adenocarcinoma of the usual type	6
	Endocervical type of adenocarcinoma	21
	Endometroid adenocarcinoma of endocervix	3
	Mucinous adenocarcinoma of endocervical type	17
Tobacco smoking history	Lifelong non-smoker	144
	Current smoker	64
	Reformed smoker > 15 years	9
	Reformed smoker ≤ 15 years	40
	Reformed smoker duration unknown	4
	NA	43
